# Low Computational Cost Distributed Acoustic Sensing Using Analog I/Q Demodulation

**DOI:** 10.3390/s19173753

**Published:** 2019-08-30

**Authors:** Fei Jiang, Zixiao Lu, Feida Cai, Honglang Li, Zhenhai Zhang, Yixin Zhang, Xuping Zhang

**Affiliations:** 1 School of Mechatronics Engineering, Beijing Institute of Technology, Beijing 100081, China; 2 Institute of Acoustics, Chinese Academy of Sciences, Beijing 100190, China; 3 National Center for Nanoscience and Technology, Beijing 100190, China; 4 Institute of Semiconductors, Chinese Academy of Sciences, Beijing 100083, China; 5 The Key Laboratory of Intelligent Optical Sensing and Manipulation, Nanjing University, Nanjing 210000, China

**Keywords:** distributed acoustic sensing, Φ-OTDR, phase demodulation, I/Q demodulation

## Abstract

Distributed acoustic sensing based on phase-sensitive optical time-domain reflectometry (Φ-OTDR) has been widely used in many fields. Phase demodulation of the Φ-OTDR signal is essential for undistorted acoustic measurement. Digital coherent detection is a universal method to implement phase demodulation, but it may cause severe computational burden. In this paper, analog I/Q demodulation is introduced into the Φ-OTDR based DAS system to solve this problem, which can directly obtain the I and Q components of the beat signal without any digital processing, meaning that the computational cost can be sharply reduced. Besides, the sampling frequency of the data acquisition card can theoretically be lower than the beat frequency as the spectrum aliasing would not affect the demodulation results, thus further reducing the data volume of the system. Experimental results show that the proposed DAS system can demodulate the phase signal with good linearity and wide frequency response range. It can also adequately recover the sound signal sensed by the optical fiber, indicating that it can be a promising solution for computational-cost-sensitive distributed acoustic sensing applications.

## 1. Introduction

Distributed acoustic sensing (DAS)—a technique that can monitor the dynamic perturbations along an optical fiber—has been widely used in many fields such as perimeter security [1,2], structure health monitoring [3], and pipeline monitoring [4,5,6,7,8]. Phase-sensitive optical time-domain reflectometry (Φ-OTDR) is the most commonly used scheme for DAS systems. It injects coherent light pulses into an optical fiber, then detects the Rayleigh backscattering (RBS) lightwave from the sensing fiber by a photodetector. External perturbations exerted on the sensing fiber can change both the amplitude and phase of the RBS lightwave. Conventional Φ-OTDR only demodulates the amplitude of the RBS lightwave to obtain the acoustic signal. However, the amplitude signal varies with the strain of the sensing fiber in a nonlinear manner, and thus may distort the retrieved acoustic signal.

By contrast, the relationship between the phase change and the fiber strain is linear. Much attention has therefore been paid to phase demodulation of RBS lightwave in recent years. Sha et al. proposed a method to extract the phase information from a direct detection based system [9], but it needs to change the pulse width according to the range of disturbance. Combining Φ-OTDR with an interferometer can also demodulate the phase signal [10,11,12,13,14,15,16], yet it increases the complexity of the system and is susceptible to environmental interference. Coherent detection is a one of the most common ways to demodulate the phase of the RBS lightwave, which has two main offshoots in the previous research. One is optical in-phase/quadrature (I/Q) demodulation [17,18,19], which uses a 90° optical hybrid to obtain the I and Q components. However, this scheme needs at least two photodetectors to detect the optical I and Q components separately, which may increase the cost of the system. The other is digital I/Q demodulation [20,21,22,23,24,25,26,27], which obtains the I and Q components in the digital domain. In all of these literatures, however, a fairly high sampling rate (at least 500 MSa/s [23]) was used to recover the beat signal, resulting in a huge amount of data. More recently, we introduced using undersampling theory to reduce the data volume of digital I/Q demodulation [28]. Nevertheless, digital I/Q demodulation needs to perform a Hilbert transform on the beat signal to obtain the I/Q components, which can still be a noticeable computational cost for the DAS system.

In this paper, a low computational cost DAS system based on coherent Φ-OTDR is presented. A 3 dB quadrature hybrid coupler is used to directly acquire the analog I and Q components according to the beat signal, meaning that we do not need to perform Hilbert transform in the digital domain anymore. Simultaneously, the I and Q components can be sampled by a low-speed data acquisition card (DAQ). In the data processing stage, we first demodulate the amplitude of the RBS lightwave. Then we locate the acoustic events according to the amplitude information. After that, we only calculate the differential phase between two points that respectively locate at the two sides of the perturbation area, rather than to demodulate the phase signals throughout the fiber. Consequently, the computational cost of the proposed DAS system is low enough to achieve real-time acoustic sensing. We also conduct a sound sensing experiment to show the availability of the proposed DAS system.

## 2. Configuration of Proposed System

**Figure 1:**
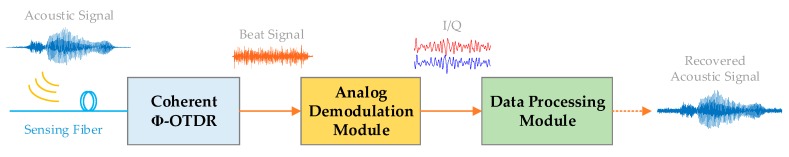
The block diagram of the proposed distributed acoustic sensing (DAS) system.

The block diagram of the proposed DAS system is depicted in Figure 1. The system consists of three main modules, including a coherent Φ-OTDR, an analog I/Q demodulation module, and a data processing module. The coherent Φ-OTDR section senses the external acoustic signals, resulting in a modulated electrical beat signal. Then the analog I/Q demodulation section splits the beat signal into I/Q components, which are then converted into digital signal by an analog–digital converter. Finally, the digital I/Q components are processed in a personal computer by the data processing module and the acoustic signal is eventually recovered. The three modules are elaborated in Section 2.1, Section 2.2 and Section 2.3, respectively.

### 2.1. Coherent Φ-OTDR

**Figure 2:**
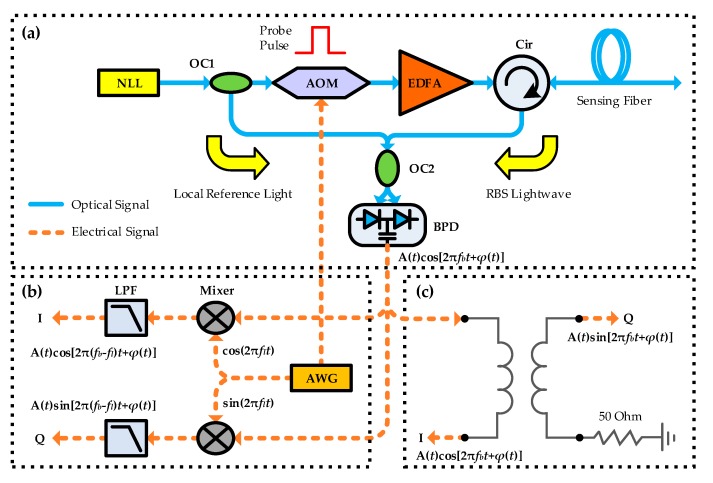
The schematic of the DAS system. (**a**) Coherent phase-sensitive optical time-domain reflectometry (Φ-OTDR); (**b**) electrical I/Q demodulator; (**c**) 3-dB quadrature hybrid coupler. NLL: narrow-linewidth laser; OC: optical coupler; AOM: acoustic–optic modulator; EDFA: erbium-doped fiber amplifier; Cir: circulator; BPD: balanced photodetector; AWG: arbitrary waveform generator.

The schematic of a typical coherent Φ-OTDR is shown in Figure 2a. A narrow linewidth laser (NLL) transmits continuous light, which is split into two streams by a 10:90 optical coupler (OC). One stream is modulated into probe pulses by an acousto–optic modulator (AOM) with a frequency shift of $f_{b}$, while the other is retained as a local reference light. After being amplified by an erbium-doped fiber amplifier (EDFA), the probe pulses are injected into the sensing fiber though a circulator. The acoustic-modulated RBS lightwave from the sensing fiber is then superposed with the local reference light at a 50:50 OC, and finally detected by a balanced photodetector (BPD). The electrical output of the BPD, also known as beat signal, can be expressed as

(1) $$I_{BPD}\left(t\right)\propto A\left(t\right)\cos\left[2\pi f_{b}t+\varphi \left(t\right)\right],$$

where A(t) and φ(t) represent the amplitude and phase of the RBS lightwave respectively. These two parameters are both modulated by the external perturbations along the sensing fiber. 

### 2.2. Analog I/Q Demodulation Module

In order to demodulate the A(t) and φ(t) in Equation (1), we need to obtain the I/Q components of the beat signal. Two schemes can be used to perform analog I/Q demodulation. One is to use an electrical I/Q demodulator, as shown in Figure 2b. One port of an arbitrary waveform generator (AWG) is used to drive the AOM, while the other serves as the local oscillator (LO) of the analog I/Q demodulator. The output beat signal from the BPD is split and respectively mixed with a pair of LO signals that are orthogonal to each other. We assume that the LO frequency is $f_{l}$, then the output of the two mixers can be expressed as:

(2) $$\left\{ \begin{array}{l} \frac{1}{2}A\left(t\right)\left\{\cos\left[2\pi \left(f_{b}-f_{l}\right)t+\varphi \left(t\right)\right]+\cos\left[2\pi \left(f_{b}+f_{l}\right)t+\varphi \left(t\right)\right]\right\}\\ \frac{1}{2}A\left(t\right)\left\{\sin\left[2\pi \left(f_{b}-f_{l}\right)t+\varphi \left(t\right)\right]-\sin\left[2\pi \left(f_{b}+f_{l}\right)t+\varphi \left(t\right)\right]\right\} \end{array}\right.$$

Low-pass filtering is then applied to suppress the high-frequency content of the mixer output. As a result, the analog I/Q components are obtained:

(3) $$\left\{ \begin{array}{l} I\left(t\right)=\frac{1}{2}A\left(t\right)\cos\left[2\pi \left(f_{b}-f_{l}\right)t+\varphi \left(t\right)\right]\\ Q\left(t\right)=\frac{1}{2}A\left(t\right)\sin\left[2\pi \left(f_{b}-f_{l}\right)t+\varphi \left(t\right)\right] \end{array}\right.$$

With respect to this scheme, an electrical LO is indispensable. Therefore, it may introduce some additional phase noise and to some extent contaminate the demodulated phase signal. 

**Figure 3:**
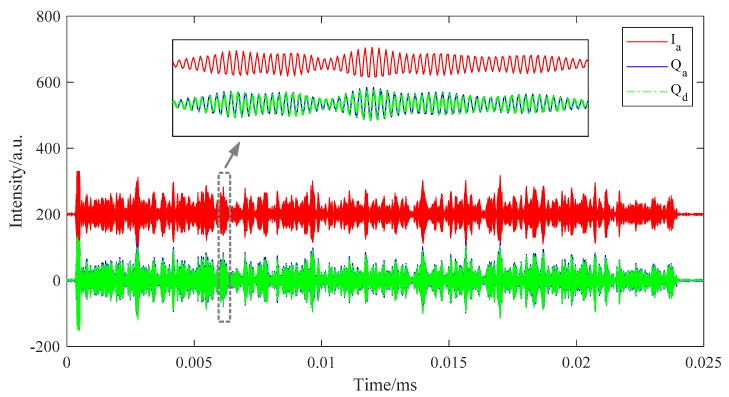
The I and Q components obtained by analog I/Q demodulation and by Hilbert transform. The curve positions are shifted for clear comparison.

The other scheme is to utilize a 3 dB quadrature hybrid coupler directly on the beat signal, as shown in Figure 2c. The beat signal can be split into two offshoots with equal intensity by the coupler: one has the same phase with the beat signal and the other has a 90 degree phase shift comparing with the beat signal. They can be regarded as the I and Q components respectively. Neither electrical LO nor LPFs are required in this scheme. Thus, it is much easier to implement than the first scheme and does not introduce extra phase noise. The second scheme is consequently adopted as the analog I/Q demodulation module in our DAS system. Figure 3 shows the analog I and Q components (represented as $I_{a}$ and $Q_{a}$ respectively) acquired by a 1 GS/s DAQ. We also plot the digital Q components (represented as $Q_{d}$) obtained by performing the Hilbert transform on $I_{a}$ here. We can see that $Q_{d}$ are basically in accordance with $Q_{a}$, which means that the analog I/Q demodulation module works pretty well. After sampling the I and Q components, we can calculate the amplitude and phase of the RBS lightwave by:

(4) $$\left\{ \begin{array}{l} A\left(t\right)\propto \sqrt{I^{2}\left(t\right)+Q^{2}\left(t\right)}\\ \varphi \left(t\right)\approx \arctan\left[\frac{Q\left(t\right)}{I\left(t\right)}\right]-2\pi f_{b}t \end{array}\right.$$

### 2.3. Data Processing

**Figure 4:**
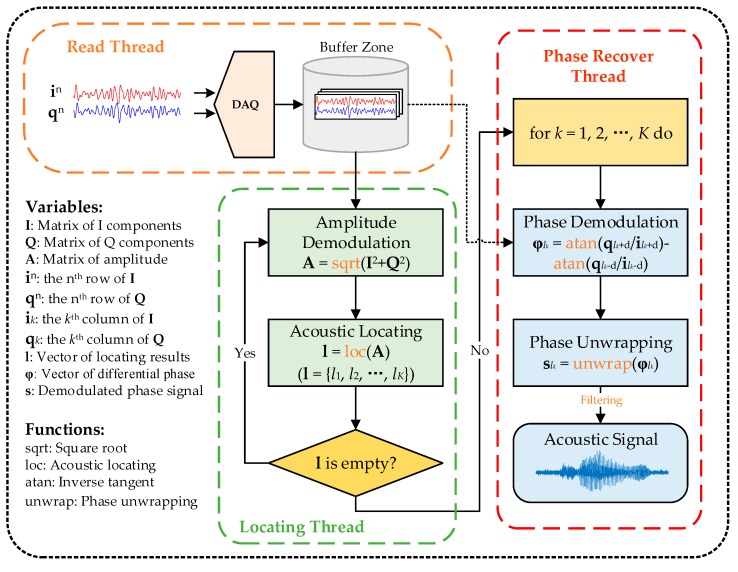
The block diagram of the data processing module.

The detail of the data processing module is depicted in Figure 4. Firstly, we save the digital I and Q components derived from the DAQ in a buffer zone as a two-dimensional queue. For ease of notation, we represent the I/Q matrix as:

(5) $$I_{N\times L}=\left[\begin{array}{cccc} i_{1}^{1} & i_{2}^{1} & \cdots & i_{L}^{1}\\ i_{1}^{2} & i_{2}^{2} & \cdots & i_{L}^{2}\\ \vdots & \vdots & \ddots & \vdots \\ i_{1}^{N} & i_{2}^{N} & \cdots & i_{L}^{N} \end{array}\right],\;Q_{N\times L}=\left[\begin{array}{cccc} q_{1}^{1} & q_{2}^{1} & \cdots & q_{L}^{1}\\ q_{1}^{2} & q_{2}^{2} & \cdots & q_{L}^{2}\\ \vdots & \vdots & \ddots & \vdots \\ q_{1}^{N} & q_{2}^{N} & \cdots & q_{L}^{N} \end{array}\right],$$

where $i_{l}^{n}$ and $q_{l}^{n}$ respectively represent the I and Q components of RBS signal at position *l* at time *n*, *N* represents the number of traces in the buffer zone, and *L* represents the number of sampling points of each trace. Meanwhile, we demodulate the amplitude of RBS lightwave according to Equation (4) in real time. Then we locate the potential acoustic events according to the amplitude matrix by a locating method. If there exists an acoustic signal at position *l_k_*, we turn back to the buffer zone and invoke the **I**/**Q** matrix to calculate the phase signal according to Equation (4). It should be noted that we only need to perform the operation at the (*l_k_* − *d*)^th^ and (*l_k_* + *d*)^th^ column of the **I**/**Q** matrix. In order to obtain the true phase induced by the acoustic signal, here *d* should be larger than half the spatial resolution of the system [26]. By phase subtraction and phase unwrapping, the phase change at the position *l_k_* can then be roughly recovered as:

(6) $$s_{l_{k}}=\mathrm{unwrap}\left[\arctan\left(\frac{q_{l_{k}+d}}{i_{l_{k}+d}}\right)-\arctan\left(\frac{q_{l_{k}-d}}{i_{l_{k}-d}}\right)\right],$$

By filtering the demodulated phase signal, the acoustic signal can finally be recovered. 

### 2.4. Computational Cost Improvement

Obviously, both digital I/Q demodulation based and analog I/Q demodulation-based DAS systems need to perform the computational process mentioned in Section 2.3. The difference between them is that analog I/Q demodulation can obtain the I and Q components without any computations, whereas digital I/Q demodulation needs massive operations. Therefore, the computational cost improvement is equal to the cost of implementing the digital I/Q demodulation.

Two methods can be used to implement digital I/Q demodulation. One is to perform the scheme presented in Figure 2b digitally. It needs to first perform element-wise multiplication between the beat signal and digital LO, and then convolve the results with an LPF. The convolution operation can be achieved by the fast Fourier transform (FFT) algorithm. The other method is to perform the Hilbert transform directly on the beat signal. Hilbert transform can also be implemented by the FFT algorithm. Therefore, the time complexity of both these methods should be O(NlogN) if the length of one RBS trace is N.

We also test the time consumption of digital I/Q demodulation by MATLAB using an Inter i7-8700 CPU. Typically, when the length of one RBS trace and the count of RBS traces are respectively 10000 and 8000, the processing time of the two digital I/Q demodulation methods are 1.12 s and 1.18 s respectively. This indicates that when using a 1 GS/s DAQ, for 1 km long fiber and 8 kHz pulse repetition frequency, digital I/Q demodulation cannot be implemented in real-time even using a premium CPU. Therefore, using analog I/Q demodulation can be a more practical scheme for a real-time DAS system.

## 3. Experimental Results

### 3.1. Experimental Setup

**Figure 5:**
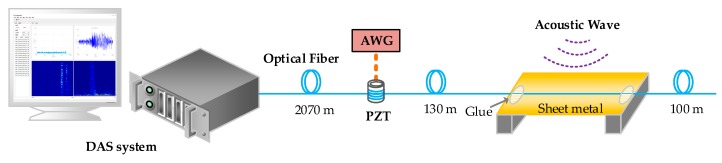
The experimental setup.

In our experimental DAS system, the optical pulse width and frequency shift induced by the AOM is 100 ns and 200 MHz respectively. The pulse repetition frequency is 8 kHz. As mentioned earlier, a 3 dB quadrature hybrid coupler is used as the analog I/Q demodulation module. A two-channel DAQ with a sampling rate of 100 MS/s is used to sample the I/Q signals. In this experiment, one piezoelectric ceramic transducer (PZT) with a 5 m fiber wound is put at approximately 2070 m over a 2300 m sensing fiber as a vibration source, which is driven by an AWG. In the vicinity of 2200 m, we pull and fix 10 cm long fiber on the surface of a sheet metal to enhance the sensitivity of acoustic wave detection, as shown in Figure 5. The validity of this structure has been proved in the previous research [29]. A loudspeaker near the sheet metal is used to produce acoustic waves. We used a female’s voice of counting from one to ten as the sound source in this experiment.

### 3.2. Results of Event Localization

**Figure 6:**
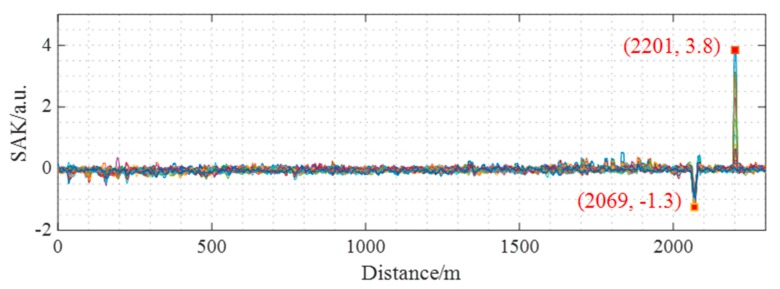
The acoustic locating result by spatial average kurtosis (SAK) method according to the amplitude information.

In our DAS system, the spatial average kurtosis (SAK) method [30] is used to locate the acoustic events. This is due to its high locating SNR and low computational cost. The locating result based on the amplitude information is shown in Figure 6. We can clearly see that the PZT vibration and the sound signal are respectively located at 2069 m and 2201 m, which means that the proposed DAS system is capable of detecting multiple acoustic events simultaneously. The SAK value of PZT vibration and the sound signal is respectively negative and positive, because the former is a harmonic signal while the latter is a percussive signal. After locating the acoustic source, the phase signal can be demodulated according to Equation (6).

### 3.3. Results of PZT Vibration

**Figure 7:**
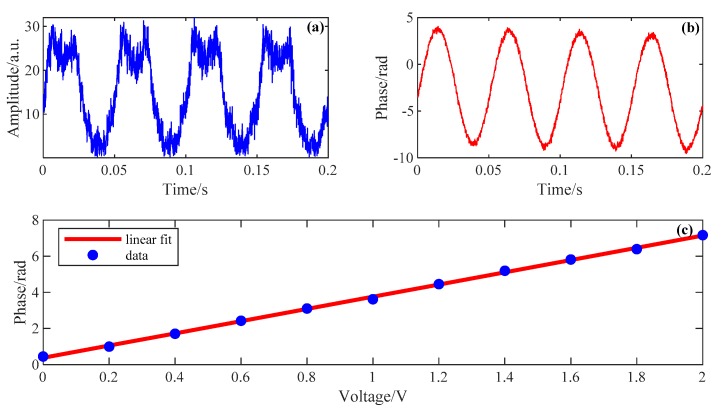
The demodulation results of PZT vibration with single-frequency. (**a**) Demodulated amplitude signal at 2070 m when 20 Hz is applied; (**b**) Demodulated phase signal at 2070 m when 20 Hz is applied; (**c**) Amplitude of the demodulated phase signal at 2070 m when different voltages are applied.

Two types of signals are applied on the PZT. First, 20 Hz single-frequency sinusoidal signals with different voltages were used to drive the PZT. Figure 7a,b respectively show the amplitude of phase demodulation results at 2070 m when the applied voltage is 2 V. We can see that the demodulated amplitude signal is severely distorted, whereas the demodulated phase signal retains high fidelity. Figure 7c shows the amplitudes of the demodulated phase signals at 2070 m when the applied voltages vary from 0 to 2 V. We can see that the proposed DAS system has a good linearity. 

**Figure 8:**
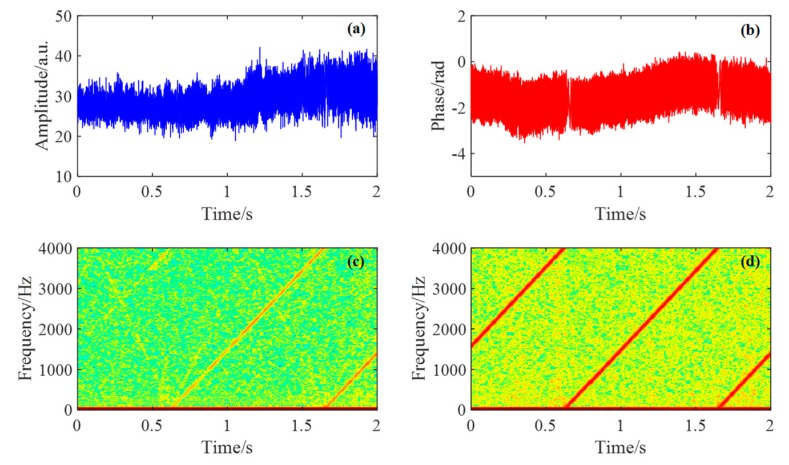
The demodulation results of piezoelectric ceramic transducer (PZT) vibration with frequency sweep. (**a**) Demodulated amplitude signal at 2070 m; (**b**) demodulated phase signal at 2070 m; (**c**) spectrogram of the demodulated amplitude signal; (**d**) spectrogram of the demodulated phase signal.

We also applied a 1 to 4000 Hz frequency sweep signal on the PZT. The demodulation results are shown in Figure 8. Figure 8a,b respectively show the demodulated time-varying amplitude and phase signal. We can see that the amplitude of demodulated amplitude signal varies with time, whereas that of the demodulated phase signal keeps nearly constant from the beginning to the end. It reveals that the sensitivity of the demodulated amplitude signal is time-varying whereas that of the demodulated phase signal is not. This is because the frequency drift of the optical source may change the interference pattern of the Rayleigh scatterers. We can also see this in Figure 8c,d, which respectively show the spectrogram of the demodulated amplitude and phase signal. 

### 3.4. Results of Sound Detection

**Figure 9:**
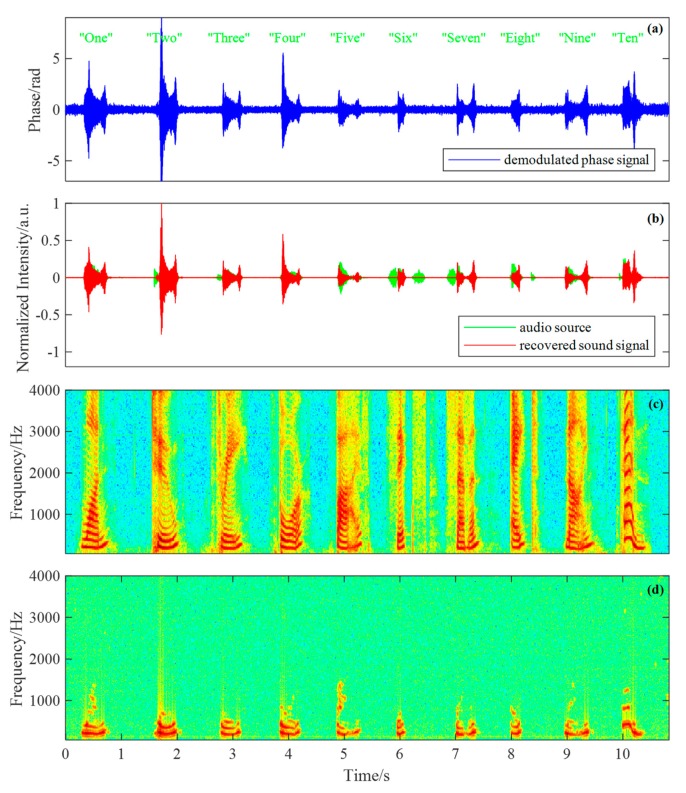
The sound sensing results at 2211 m. (**a**) The demodulated phase signal; (**b**) the audio source signal and the recovered sound signal; (**c**) the spectrogram of the audio source signal; (**d**) the spectrogram of the recovered sound signal.

Figure 9a shows the demodulated time-varying phase signal at 2200 m, which is filtered by a 50 Hz high-pass digital filter in order to remove the trend term of the signal. We can see that the demodulated phase signal is noisy to some extent. It is mainly contaminated by white noise, which is on account of the system noise including phase noise of light source, partial interferometric problem, and electrical noises such as thermal noise and shot noise [31].

Spectral subtraction method [32] is used in our DAS system to reduce the noise of the demodulated phase signal, resulting in the recovered sound signal. The green line and red line shown in Figure 9b respectively represent the audio source signal and the recovered sound signal. We can see that most of the utterances are well recovered except the phoneme /s/ and /t/. More signal details can be seen in Figure 9c,d, which respectively show the spectrogram of the audio source signal and the recovered sound signal. We can see that the high frequency components of the recovered sound signal tend to be obscure. This is caused by the non-uniform frequency response of the acoustic sensing structure shown in Figure 5. Despite that, the ten utterances can still be clearly recognized from the recovered sound signal. The recovered sound files are available in the Supplementary Material.

## 4. Conclusions

In this paper, we introduced using analog I/Q demodulation to achieve phase measuring for the Φ-OTDR based DAS system. The proposed DAS system can not only use a lower-speed DAQ, but also bypass the digital I/Q computing process. As a result, the computational cost of the proposed system becomes much lower than the previous DAS systems based on digital coherent detection. The Q component obtained by the analog I/Q demodulation module is proved to be virtually consistent with that obtained by the Hilbert transform. We also conducted the PZT vibration sensing and sound sensing experiments. The experimental results show that the proposed DAS system can demodulate the phase signal with good linearity and wide frequency response range and can well recover the sound signal sensed by the optical fiber. In conclusion, the proposed DAS system is promising for practical applications due to its capability of phase measuring and low computational cost.

## Supplementary Materials

The following are available online at https://www.mdpi.com/1424-8220/19/17/3753/s1, Audio S1: the audio source used in the experiment, Audio S2: the demodulated phase signal, Audio S3: the recovered sound signal.
